# The *APOE*^ε3/ε4^ Genotype Drives Distinct Gene Signatures in the Cortex of Young Mice

**DOI:** 10.3389/fnagi.2022.838436

**Published:** 2022-03-16

**Authors:** Kate E. Foley, Amanda A. Hewes, Dylan T. Garceau, Kevin P. Kotredes, Gregory W. Carter, Michael Sasner, Gareth R. Howell

**Affiliations:** ^1^The Jackson Laboratory, Bar Harbor, ME, United States; ^2^School of Graduate Biomedical Sciences, Tufts University School of Medicine, Boston, MA, United States; ^3^Department of Psychology, University of Maine, Orono, ME, United States; ^4^Graduate School of Biomedical Sciences and Engineering, University of Maine, Orono, ME, United States

**Keywords:** APOE4 and APOE3, APOE4 and AD risk, transcriptome (RNA-seq), exercise, Alzheimer’s disease, dementia - Alzheimer’s disease, dementia, cerebral cortex

## Abstract

**Introduction:**

Restrictions on existing *APOE* mouse models have impacted research toward understanding the strongest genetic risk factor contributing to Alzheimer’s disease (AD) and dementia, *APOE^ε4^*, by hindering observation of a key, common genotype in humans – *APOE^ε3/ε4^*. Human studies are typically underpowered to address *APOE^ε4^* allele risk as the *APOE^ε4/ε4^* genotype is rare, which leaves human and mouse research unsupported to evaluate the *APOE^ε3/ε4^* genotype on molecular and pathological risk for AD and dementia.

**Methods:**

As a part of MODEL-AD, we created and validated new versions of humanized *APOE^ε3/ε3^* and *APOE^ε4/ε4^* mouse strains that, due to unrestricted breeding, allow for the evaluation of the *APOE^ε3/ε4^* genotype. As biometric measures are often translatable between mouse and human, we profiled circulating lipid concentrations. We also performed transcriptional profiling of the cerebral cortex at 2 and 4 months (mos), comparing *APOE^ε3/ε4^* and *APOE^ε4/ε4^* to the reference *APOE^ε3/ε3^* using linear modeling and WGCNA. Further, *APOE* mice were exercised and compared to litter-matched sedentary controls, to evaluate the interaction between *APOE^ε4^* and exercise at a young age.

**Results:**

Expression of human APOE isoforms were confirmed in *APOE^ε3/ε3^, APOE^ε3/ε4^* and *APOE^ε4/ε4^* mouse brains. At two mos, cholesterol composition was influenced by sex, but not *APOE* genotype. Results show that the *APOE^ε3/ε4^* and *APOE^ε4/ε4^* genotype exert differential effects on cortical gene expression. *APOE^ε3/ε4^* uniquely impacts ‘hormone regulation’ and ‘insulin signaling,’ terms absent in *APOE^ε4/ε4^* data. At four mos, cholesterol and triglyceride levels were affected by sex and activity, with only triglyceride levels influenced by *APOE* genotype. Linear modeling revealed *APOE^ε3/ε4^*, but not *APOE^ε4/ε4^*, affected ‘extracellular matrix’ and ‘blood coagulation’ related terms. We confirmed these results using WGCNA, indicating robust, yet subtle, transcriptional patterns. While there was little evidence of *APOE* genotype by exercise interaction on the cortical transcriptome at this young age, running was predicted to affect myelination and gliogenesis, independent of *APOE* genotype with few *APOE* genotype-specific affects identified.

**Discussion:**

*APOE^ε4^* allele dosage-specific effects were observed in circulating lipid levels and cortical transcriptional profiles. Future studies are needed to establish how these data may contribute to therapeutic development in *APOE^ε3/ε4^* and *APOE^ε4/ε4^* dementia patients.

## Introduction

The ε*4* allele of apolipoprotein E (*APOE*), *APOE^ε4^*, has been identified as one of the greatest genetic risk factors for Alzheimer’s disease (AD) and related dementias (ADRDs) (Rohn, 2014; Halliday et al., 2016; Riedel et al., 2016; Koizumi et al., 2018; Zhao et al., 2020). The ε*3* and ε*2* alleles of *APOE* confer neutral and protective risk, respectively. In late-onset AD (LOAD), allele frequencies vary between cases and controls, with *APOE^ε2^*, *APOE^ε3^*, and *APOE^ε4^* present at a rate of 8%, 78%, and 14%, respectively, in unaffected subjects, and 4%, 59% and 37%, respectively, in affected subjects (Farrer et al., 1997). The frequency of LOAD increases from 20% in a non-carrier of *APOE^ε4^*, to 47% when carrying one copy, and up to 91% when carrying two copies (Bell et al., 2012). In VaD, *APOE^ε4^* predisposes individuals for increased risk of cerebrovascular disease and ischemic stroke, potentially up to 30% (Belloy et al., 2019). With *APOE^ε4^* being less frequent than *APOE^ε3^*, the more common risk genotype in at risk populations is *APOE^ε3/ε4^*, with 41.1% of LOAD cases possessing the *APOE^ε3/ε4^* genotype compared to 14.8% with *APOE^ε4/ε4^* (Farrer et al., 1997).

Genetic variation between mouse strain *Apoe* has been proposed to modify phenotypes relevant to human aging and dementia (Neuner et al., 2019). However, mouse strains do not carry equivalent *Apoe* alleles to those observed in humans. Therefore, humanized *APOE* mice have contributed greatly to what we currently understand about the myriad of APOE mechanisms that may contribute risk for dementia. *APOE^ε4^* is predicted to increase risk for multiple dementias through either gain of toxic function or loss of function which is context dependent (Belloy et al., 2019). Additionally, it is well understood that there are at least two major compartments by which APOE functions –peripherally (i.e., blood) and centrally (i.e., brain) (Chan et al., 1989; Hauser et al., 2011; Chernick et al., 2019). Peripheral APOE (also referred to as circulating APOE), produced primarily by liver hepatocytes, remains in the blood and circulatory system, and is thought to not cross the blood brain barrier (Liu et al., 2012). A primary role of circulating APOE is in lipid and cholesterol homeostasis with APOE functioning as a lipid trafficking protein. The lipid binding region and receptor binding region differ in affinity to its respective receptors between *APOE^ε2^*, *APOE^ε3^*, and *APOE^ε4^*. Cerebral APOE is produced mainly by astrocytes, however during stress can be upregulated and produced by microglia and possibly neurons (Chan et al., 1989; Hauser et al., 2011; Chernick et al., 2019). Studies have utilized humanized *APOE* mouse models crossed with amyloid and tau mouse models to show that, in addition to lipid trafficking, APOE functions in a variety of AD-relevant processes including amyloid clearance and tau-mediated neurodegeneration (Sullivan et al., 1997; Knouff et al., 1999; Huang et al., 2017; Liu et al., 2017; Shi et al., 2017; Huynh et al., 2019). However, these studies assessed homozygous *APOE^ε3/ε3^* and *APOE^ε4/ε4^*, but not the heterozygous *APOE^ε3/ε4^* genotype.

Human and murine studies have parsed out important mechanisms by which the *APOE^ε4^* allele differs in comparison to the *APOE^ε3^* allele to increase detrimental brain pathology leading to AD and other dementias. However, little is known about the mechanisms by which *APOE^ε3^* and *APOE^ε4^* may interact in those with the *APOE^ε3/ε4^* genotype to affect risk for dementia. This lack of knowledge has been in part due to legal restrictions on the breeding of *APOE^ε4/ε4^* to *APOE^ε3/ε3^* mice to create *APOE^ε3/ε4^* mice. This limitation has also meant that experiments comparing *APOE^ε3/ε3^* to *APOE^ε4/ε4^* were often not performed in litter-matched mice. It has been shown that the APOE^ε4^ isoform is degraded at a higher rate by astrocytes compared to the APOE^ε3^ isoform (Ramaswamy et al., 2005; Riedel et al., 2016). Others have shown differential bioenergetics in male *APOE^ε3/ε4^* mice compared to male *APOE^ε3/ε3^* mice (Area-Gomez et al., 2020). Nonetheless, much is still to be learned about the global consequences of the *APOE^ε3/ε4^* genotype. It is conceivable there may be compensatory, dominant, or *APOE^ε4^* dose-dependent effects on molecular changes predisposing risk for ADRDs.

To improve translatability of *APOE* mouse models to enable new discoveries for human biology, here we describe the creation of a new set of humanized *APOE* mouse models, using a similar design to models commonly used (Sullivan et al., 1997; Knouff et al., 1999). There are no breeding or distribution restrictions on these new humanized *APOE* models. Further, we used our new humanized *APOE* mouse models to test our hypothesis that *APOE^ε3/ε4^* mice show characteristics that may modify risk for dementias that are distinct from *APOE^ε4/ε4^* mice. First, we assessed male and female *APOE* mice, examining the effects of sex and *APOE^ε4^* at 2 months (2 mos) of age. We next evaluated a cohort at 4 months (4 mos) which also including a running cohort to evaluate the potential interactions between *APOE^ε4^* and exercise. Lipid profiling was performed on plasma samples while RNA-seq was performed on the cerebral cortex. Linear modeling, a mathematical approach widely used for analyzing transcriptomic data in both humans and mice, was used identify the contribution of main and interacting factors (genotype, sex, activity) on the cortical transcriptome (Wang and Brinton, 2016; Belloy et al., 2019; Williams et al., 2020). Results revealed significant differences between the *APOE* genotypes in both the periphery and the cortex supporting our hypothesis that the *APOE^ε3/ε4^* genotype exerts unique effects compared to the *APOE^ε4/ε4^* genotype.

## Materials and Methods

### Mouse Husbandry

All experiments involving mice were conducted with approval and accordance described in the Guide for the Care and Use of Laboratory Animals of the National Institutes of Health. All experiments were approved by the Animal Care and Use Committee at The Jackson Laboratory. Mice were kept in a 12/12-h light/dark cycle and fed *ad libitum* 6% kcal fat standard mouse chow.

### Creation of Humanized Apolipoprotein E Mouse Strains

**FIGURE 1:**
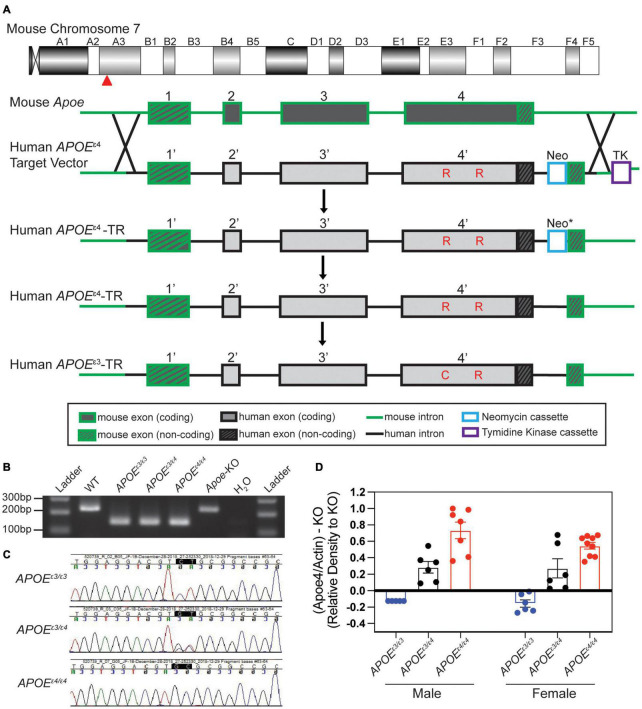
Creation and validation of *APOE* allelic series. **(A)** Schematic of human *APOE* allele insertion at the mouse *Apoe* locus on chromosome 7. Exon 1 remained unchanged (mouse) and exons 2–4 were replaced with human *APOE^ε4^* sequence through homology directed repair (HDR). The neomycin cassette was removed (* indicated by asterisk) through Flp-Frt breeding (Flp-Frt sites not shown). The *APOE^ε3^* SNP was changed through CRISPR mediated endonuclease activity. Critical amino acids differing between *APOE^ε4^* and *APOE^ε3^* noted in red. **(B)** PCR based assay to identify humanized *APOE* insertions at exon 2 vs. WT mouse *Apoe* (no insertion) at exon 2. **(C)** Sanger sequencing to distinguish *APOE^ε3^* and/or *APOE^ε4^* alleles. **(D)** Western blotting revealed significant differences in APOE^ε4^ levels between genotypes.

Humanized *APOE* mice were created in collaboration with the Genetic Engineering Technologies core at The Jackson Laboratory. The mouse *Apoe* gene is located on chromosome 7 at 19,696,109–19,699,166. An *APOE^ε4^* gene-targeting construct was made that included 4980 bp of mouse sequence, which defined the mouse 5’ homology arm including exon 1 of mouse *Apoe*, 4292 bp of human *APOE^ε4^* sequence including human protein coding exons 2–4 of the human gene as well as an additional 1.5 kb of flanking human sequence after the 3′UTR to include any potential regulatory sequences. Exon 4 contained sequence that encoded the *APOE^ε4^* isoform (nucleotide sequence for arginine at R130 and R176). The predicted protein sequence in our humanized *APOE^ε4^* mouse model is 317 amino acids and includes the18 amino acid signal peptide at the N-terminus. As in all *APOE* isoforms, the 18 amino acid leader sequence is cleaved resulting in the mature 299 amino acid protein, with the amino acids that define *APOE^ε4^* located at positions 112 and 158 (Mahley and Rall, 2000; Rohn and Moore, 2017). A Frt-neo-Frt (FNF) selection cassette was inserted after the human sequence followed by a Nde1 restriction site (for ease of Southern screening). The FNF cassette was followed by 5166 bp of mouse sequence, the 3’ homology arm. The resulting 14,438 bp synthesized construct was cloned into pBlight vector using recombineering techniques, producing a construct called mApoE_hAPOE4_PGKneo_mAPOE for gene targeting in embryonic stem cells. The *APOE^ε4^* gene-targeting construct was introduced into cultured embryonic stem (ES) cells of a C57BL/6J (B6) mouse strain by electroporation. Homologous recombination produced loci that retained all normal mouse regulatory sequences (plus non-coding exon one) together with the human APOE^ε4^ protein-encoding exons 2–4 (Figure 1A). Transfected ES cells were screened by Southern blot to ensure correct targeting. Three clones were identified that were correctly targeted. ES cells containing the correctly targeted locus were introduced into albino B6 embryos, and the resultant chimeric mice were bred with B6 mice. Offspring carrying the modified locus in the germline were interbred to generate the homozygous genetically modified genome. All F1 matings produced normal litter sizes with a Mendelian distribution of the locus. Heterozygous animals were crossed to FLP recombinase expression mice (JAX Stock No. 005703) to remove the FRT site flanked PGK-neo cassette. Mice that no longer contained the FRT flanked PGK-neo cassette were then backcrossed to B6 at least once to remove the FLP recombinase transgene. SNP analysis was performed to validate the B6 background. Offspring that were negative for the FLP recombinase transgene were then interbred and maintained as *APOE^ε4/ε4^* homozygous mice (JAX Stock No. 000664).

The *APOE^ε3^* model was generated using CRISPR/Cas9-mediated gene targeting in *APOE^ε4^* zygotes (Figure 1A). An *APOE^ε4^* specific sgRNA (GCGGACATGGAGGACGTGCG CGG; PAM site is underlined) was used to target the human knock-in allele just downstream of the valine codon that defines the *APOE^ε4^* allele, and a 129 nt single stranded oligonucleotide (GAGACGCGGGCACGGCTGTCCAAGGAGCTGCAGGCGG CGCAGGCCCGGCTGGGCGCGGACATGGAGGACGTc**T**GC GGCCGCCTGGTGCAGTACCGCGGCGAGGTGCAGGCCA TGCTCGGCCA) including the CGC->**T**GC substitution (bold) to change arginine to cysteine and silent mutation GTG-GTC (underlined) to prevent re-cutting. Putative founders were bred to B6 mice for at least four generations and then interbred to be maintained as *APOE*^ε3/ε3^ homozygous mice (JAX Stock No. 029018).

To create experimental cohorts, *APOE^ε3/ε3^* mice were crossed to *APOE^ε4/ε4^* mice to generate *APOE^ε3/ε4^* mice, which were then intercrossed to give cohorts of litter-matched mice of all required genotypes (*APOE^ε3/ε3^*, *APOE^ε3/ε4^*, and *APOE^ε4/ε4^*). Three separate cohorts of mice were used in this study. The first was aged to 2 mos for strain validation including western blotting. The second was aged to 2 mos for peripheral and cortical assessments. The third was aged to 1 mo, when half were provided a running wheel, and subsequently assessed at 4 mos along with sedentary controls.

### Genotyping of Apolipoprotein E Mice

Genotyping to differentiate between *APOE^ε3^* and *APOE^ε4^* alleles for our experimental cohorts was performed *via* ear punch at 1 mo of age. A gel-based PCR assay was used to determine insertion of the humanized construct (Figure 1B). Primers included a common forward spanning over intron 1 and exon 2 (AATTTTTCCCTCCGCAGACT), a wild type (WT) mouse reverse in intron 2 (ACAGCTGCTCAGGGCTATTG), and a humanized reverse (AGGAGGTTGAGGTGAGGATG). A band for WT (wild type – mouse control, no humanized insertion) shows at 244 bp, while the humanized insertion results in a 148 bp band. To differentiate between the *APOE^ε3^* and *APOE^ε4^* alleles, Sanger sequencing was used to identify CT for *APOE^ε3^*, and GC for *APOE^ε4^*, and a double peak signifying the presence of both *APOE^ε3^* and *APOE^ε4^* (GT) (Figure 1C).

### Validation of Apolipoprotein E Expression by Western Blotting

Snap-frozen hemispheres were homogenized by hard tissue homogenizer (USA Scientific, Ocala, FL, United States) and lysed in 700 mL RIPA buffer (R0278, Sigma, St. Louis, MO, United States) supplemented with 100× protease and phosphatase inhibitor reagents (1861281, Thermo Fisher Scientific, Waltham, MA, United States). Lysates were incubated for 1 h at 4°C before pelleting insoluble proteins by spinning at 4°C, 11,000 × *g* for 15 min. Protein concentration was determined by Bradford protein assay (Biorad, Hercules, CA, United States), according to manufacturer’s instructions. Samples were mixed with 10× Laemlli buffer (42556.01, Amsbio, Cambridge, MA, United States), boiled for 10 min, and run on 12% SDS PAGE gels (456-1044, BioRad) with colorimetric ladder (RPN800E, GE, Boston, MA, United States). Gels were transferred to PVDF membranes for immunoblotting and imaging using an iBlot2 dry blotting system (Thermo Fisher). Membranes were blocked in 5% non-fat dry milk in 1XPBS + 0.1% Tween20 for 1 h prior to incubating with primary antibodies diluted in 5% non-fat dry milk in 1XPBS + 0.1% Tween20 for 1 h at room temperature. Membranes were washed in 1XPBS + 0.1% Tween20 before incubating with secondary antibodies diluted in 5% non-fat dry milk in 1XPBS + 0.1% Tween20. HRP-conjugated secondary antibodies targeting primary antibody host IgG were incubated at 1 h at room temperature. Membranes were washed in 1XPBS + 0.1% Tween20 before digital imaging with SuperSignal West Pico PLUS chemiluminescent substrate (34579, Thermo Fisher). Antibodies used include: Pan human APOE (AB947, Millipore, Burlington, MA, United States); Mouse Apoe (NB100-240, Novus Biologicals, Centennial, CO, United States); human APOE^ε4^ (NBP1-49529, Novus Biologicals); Actin (ab179467, Abcam, Cambridge, United Kingdom).

For APOE^ε4^ quantification, western blots were run as stated above with modifications. Protein extraction was performed on snap frozen cerebral cortex tissue in 700–1000 mL RIPA buffer supplemented with cOmplete Mini Protease Inhibitor Cocktail (Millipore Sigma, 11836153001), 100 uM PMSF, and 100 μM Na3V4O. Protein was quantified by Bradford Protein Assay as stated above. Samples were mixed with 2× Laemmli buffer and RIPA buffer to dilute all proteins to 100 μg/μl/sample. Samples were heated for 5 min at 95°C, and run on 4–20% gradient gels (BioRad, 4561096) with Full Range Rainbow Recombinant Protein Molecular Marker ladder (RPN800E). Gels were transferred to nitrocellulose membranes (Thermo Fisher, IB301001) using the iBlot system (Thermo Fisher) for immunoblotting. Membranes were blocked in 5% non-fat dry milk in 1XPBS + 0.1% Tween20 for 1 h. Primary antibodies were diluted in 5% non-fat dry milk in 1XPBS + 0.1% Tween20 and incubated overnight at 4°C moving. Membranes were washed in 5% non-fat dry milk in 1XPBS + 0.1%Tween20 and then incubated. Secondary antibodies were also diluted in 5% non-fat dry milk in 1XPBS + 0.1% Tween20 for 2 h at room temperature. Membranes were washed again in 5% non-fat dry milk in 1XPBS + 0.1%Tween20 and exposed to Amersham ECL Western Blotting Detection Reagent (RPN2109, Cytiva, Marlborough, MA, United States) just prior to digital imaging on the Azure Biosystems c600 (Azure Biosystems, Dublin, CA, United States). Actin and APOE^ε4^ were quantified in ImageJ (FIJI).

### Exercise by Voluntary Running

At first, mice were group housed (two or three per pen) and were provided access to low profile saucer wheels (Innovive Inc.) 24 h a day from 1 mo to 4 mos. Sedentary mice were not provided access to running wheels. At 4 mos, just prior to harvest, mice were separated and individually housed with a trackable low-profile running wheel (Med Associates Inc.) or no wheel access. Rotations per minute during lights out (12 h, 6:00 pm – 6:00 am) were quantified. This time period was used as it is when mice are naturally awake and active. Running wheel rotations were measured in 1-min bins to allow for distance traveled (sum of rotations) calculated per mouse each night. Average rotations were calculated per mouse. Average speed while active was calculated by isolating the minute intervals where activity was measured (>0) and averaging the number of rotations for the minutes active. Percent of time at each speed was calculated by totaling the number of minute bins that mice ran between 0, 1–30 rotations, 31–70 rotations, 71–100 rotations, and 100+ rotations and dividing by the total amount of minutes tracked. Any nights that had fewer than 700 min tracked were excluded from analysis.

### Harvesting, Tissue Preparation and Blood Lipid Profiling Assessment

All mice were euthanized by intraperitoneal injection of a lethal dose of Ketamine (100 mg/ml)/Xylazine (20 mg/ml) and blood was collected in K2 EDTA (1.0 mg) microtainer tubes (BD, Franklin Lakes, NJ, United States) through approved cardiac puncture protocols. Mice were perfused intracardially with 1XPBS. Brains were carefully dissected then hemisected sagittally, and the cortex (Ctx) was then carefully isolated and snap frozen in solid CO_2_ for RNA-sequencing and cholesterol profiling. Blood was kept at room temperature for at least 30 min to prevent clotting, and then centrifuged at 21°C for 10 min at 5000 rpm. Plasma was carefully collected. Plasma and brain lipid concentrations were characterized on the Beckman Coulter AU680 chemistry analyzer.

### RNA Extraction, Library Construction, RNA Sequencing, and RNA Sequencing Quality Control

RNA sequencing (RNA-seq) was performed by The Jackson Laboratory Genome Technologies Core. RNA extraction involved homogenization with TRIzol (Invitrogen, Waltham, MA, United States) as previously described (Soto et al., 2015). RNA was isolated and purified using the QIAGEN miRNeasy mini extraction kit (Qiagen, Hilden, Germany) in accordance with manufacturer’s instructions. RNA quality was measured *via* the Bioanalyzer 2100 (Agilent, Santa Clara, CA, United States) and poly(A) RNA-seq sequencing libraries were compiled by TruSeq RNA Sample preparation kit v2 (Illumina, San Diego, CA, United States). Quantification was performed using qPCR (Kapa Biosystems). RNA-seq was performed on the HiSeq 4000 platform (Illumina) for 2 × 100 bp reads for a total of 45 million reads according to the manufacturer’s instructions. Quality control for each sample was completed using NGSQCToolkit v2.3 which removed adaptors and trimmed low quality bases (Phred < 30) (Patel and Jain, 2012). To quantify gene expression of the trimmed reads, we used RSEM v1.2.12 which uses Bowtie2 v2.2.0 for alignment of these reads (Langmead et al., 2009). We used a custom mouse genome including mm-10 based upon the B6 reference genome, with the addition of the human *APOE* gene for continuity.

### Linear Modeling and Functional Enrichment

Genes were filtered by (1) removing all genes that did not vary in expression (gene count change across all samples was 0) and (2) removing all genes that did not have at least five reads in 50% of the samples. Remaining genes were normalized using DEseq2 (Love et al., 2014). Variance stabilizing transformation (vst) was then performed. Principal component analysis (PCA) identified three outliers from the 2 mo dataset, and five outliers from the 4 mo dataset which were excluded from further analysis. *APOE* genotype will be referred to as ‘genotype.’ A linear model was used on the normalized counts to identify genes significantly fit by the predictors of our model: sex, genotype, and sex-genotype in the 2 mo dataset, and sex, genotype, activity, and genotype-activity in the 4 mo dataset. Reference data in the model were ‘female *APOE^ε3/ε3^’* for 2 mo and ‘female sedentary *APOE^ε3/ε3^*, for 4 mo data. The linear model was performed on 18,402 genes for 2 mos and on 18,987 genes for 4 mos. Nominal significant genes were identified per predictor term (sex, genotype, etc.) based upon Pr(>|t|< 0.05). Functional enrichment for each linear model predictor term was performed using the clusterProfiler package with *p* < 0.10 to determine significant GO terms (Yu et al., 2012). Bonferroni Hochberg (BH) conditions were chosen for pAdjustMethod. Background lists consisted of all genes prior to significant geneset filtering.

### Comparison of *APOE^ε3/3^* and *APOE^ε4/4^* Samples to Zhao et al.

Previously published cortical transcriptional data was accessed through the AD Knowledge Portal. Gene expression from Zhao et al. (2020) utilized conditional quantile normalization (CQN)-normalized log2RPKM values. Samples from 3 mo mice were filtered for, and then used for PCA clustering. To match previous publications, no scaling or centering was used in this PCA. Linear modeling was run on 19,120 genes across 32 samples with factors for sex, genotype, and sex-genotype. Significant genes were identified per predictor term (sex, genotype, etc.) based upon Pr(> | t| < 0.05). Functional enrichment for each linear model predictor term was performed using the clusterProfiler package with *p* < 0.05 to determine significant GO terms (Yu et al., 2012).

### Cell Type Specific Enrichment

Publicly available cell type gene expression was downloaded from the brainrna-seq.org dataset generated by the Barres laboratory (Zhang et al., 2014, 2016). Significant genes from linear model results from 2- and 4- mo data were cross referenced to this dataset. Some genes in our datasets were not found in the brainRNA-seq dataset and were excluded. Number of genes per cell type was expressed as a percentage of a specific cell type divided by all genes that were able to be cross referenced for the linear model predictor terms.

### Weighted Gene Co-expression Network Analysis

Weighted gene co-expression network analysis required the WGCNA package by Horvath and Langfelder (Langfelder and Horvath, 2008, 2012). Normalized and variance transformed data (vst) without outliers was used to build weight gene co-expression networks. First, all samples passed the function goodSamplesGenes to check for incomplete sample data. Samples were clustered to identify outliers using hierarchical clustering; two samples were excluded from further analysis. Next, the soft-thresholding power (b) was chosen by calculating scale-free topology through the relationship between power and scale independence which resulted in a softPower of 5. Then 18 modules were clustered based upon minModuleSize = 30, and a mergeCutHeight = 0.4. These values were chosen based off previous studies with similar methods to achieve non-redundant smaller modules (Zhao et al., 2020). Module eigengenes for each module were identified and the correlation to sex, genotype, and activity was computed. The p values were then corrected for multiple testing both within and across terms by calculating false discovery rates. Genes in each module were annotated by using the AnnotationDbi package (Hervé Pagès et al., 2020). Module membership (module eigengene correlated with gene expression) was regressed against gene significance (the correlation between the linear model predictor term and each individual gene) to identify highly correlated modules (Pearson correlation). Genes in a module were processed through clusterProfiler to identify functional enrichment.

### Neuronal Counts

To quantify neuronal cell number, 4 mo female sedentary mice were evaluated as previous studies would suggest they would be the most susceptible to neuronal loss. Two of each genotype (six samples total) were stained in replicates of three (three brain slices) and then evaluated for NEUN+ DAPI+ cells in the cortex above the CA1 in the hippocampus. Brains were cryosectioned at 20 mm onto slides. Sections were blocked for 1 h in 2%PBT + 10% Normal Goat Serum. Primary antibody NEUN (abcam, ab104225) was diluted in 2% PBT + 10% Normal Goat Serum and kept at 4°C overnight. Slides were washed 3 × 10 min in 2% PBT. Secondary antibody (goat anti rabbit 488) was diluted in 2%PBT and kept for 3 h at room temperature. A representative cortical section was imaged on a Leica SP8 confocal microscope at 1 um step size for 20 um. Images were quantified identically with the spots feature through IMARIS software for NEUN+ and DAPI+ cells.

### Statistical Analysis

Statistical analysis for 2 mo biometric data including cholesterol composition, triglyceride, unfasted glucose, and non-esterified fatty acids (NEFA) were calculated by two-way ANOVA (interactions tested: sex-genotype, main effects: sex, genotype), and a *post hoc* Tukey test in GraphPad Prism v7.0a. Outliers were identified by ROUT (*Q* = 1.0%) using GraphPad Prism v7.0a. Statistical analysis for 4 mo biometric data was performed by three-way ANOVA (interactions tested: sex-genotype-activity, genotype-activity, sex-activity, sex-genotype, main effects tested: sex, activity, genotype) followed by a TukeyHSD *post hoc* test in R v1.2.1335. Terms were considered significant if *p* < 0.05. *Post hoc* tests were used to determine differences between genotypes per sex, significant effects between genotypes and sexes were not reported (i.e., female-sed-*APOE^ε3/ε4^* to male-sed-*APOE^ε3/ε3^*). All weight and biometric data included groups of 5–16 mice per sex/genotype/activity. Transcriptional profiling was performed on groups of six mice per sex/genotype/activity. Details of statistical analyses of linear modeling, functional enrichment, and WGCNA data are provided above.

## Results

### Creation of Humanized *APOE^ε3/ε3^*, *APOE^ε3/ε4^*, and *APOE^ε4/ε4^* Mice

Humanized *APOE^ε3/ε3^* and *APOE^ε4/ε4^* mice were created on C57BL/6J (B6) mouse strain using a similar design to that described previously (Supplementary Figure 1A, see the section “Materials and Methods”) (Sullivan et al., 1997; Knouff et al., 1999). Briefly, exons two through four of the mouse *Apoe* gene were replaced by sequence encoding the equivalent region in human the *APOE^ε4^* gene through homologous directed repair (HDR) (Figure 1A and Supplementary Figures 1B,C). The *APOE^ε3^* allele was produced by CRISPR-Cas9 of *APOE^ε4/ε4^* mice (Figures 1B,C). A PCR-based assay combined with Sanger sequencing was used to confirm humanized *APOE* alleles (Figures 1B,C). Protein was confirmed by western blotting with human APOE protein present in all humanized APOE mice. APOE^ε4^ protein only detectable in *APOE^ε3/ε4^* and *APOE^ε4/ε4^* mice in a relative abundance of 0.38 and 0.49, respectively (Figure 1D and Supplementary Figures 2,3).

**FIGURE 2:**
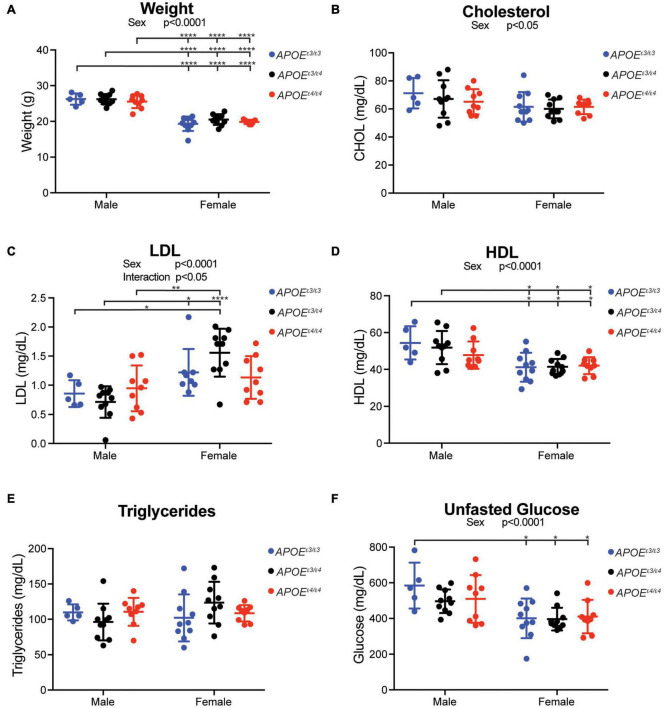
Plasma cholesterol composition affected by sex, but not *APOE* genotype, at 2 months. **(A)** Weight (grams, g) at harvest in both male and female *APOE* mice showed expected significant sex differences across all genotypes. **(B)** Total plasma cholesterol concentration revealed a significant effect of sex. **(C)** Low density lipoprotein (LDL) concentration showed a significant interactive effect between *APOE* genotype and sex, as well as a main effect of sex. **(D)** High density lipoprotein concentration (HDL) showed a significant effect of sex. **(E)** Triglyceride concentration showed no effect by *APOE* genotype or sex. **(F)** Unfasted glucose showed a significant sex effect. Significant ANOVA results are stated under the title of the graph, significant Tukey *post hoc* results are shown on graph between significant groups. All significance data can be found in Supplementary Table 1.

To evaluate whether *APOE^ε4^* dosage affected body weight and circulating lipids, two translatable biometric measurements, litter-matched male and female *APOE^ε3/ε3^*, *APOE^ε3/ε4^* and *APOE^ε4/ε4^* mice were assayed at 2 mos. As expected, body weight was significantly different between sexes, with males weighing more than females. However, body weight was not affected by *APOE* genotype (Figure 2A). Total cholesterol, HDL, and unfasted glucose levels in plasma showed a significant main effect of sex, with males showing higher concentrations than females (Figures 2B,D,F). There was a significant interaction between sex and *APOE* genotype for LDL with the sexes showing differing trends in *APOE^ε3/ε4^* mice (Figure 2C). Plasma triglyceride levels, and brain total cholesterol, HDL, and LDL concentrations did not show an effect of sex or *APOE* genotype (Figure 2E and Supplementary Figure 4).

### Cortical Transcriptional Profiling Reveals Unique Effects of *APOE^ε3/ε4^* Genotype at 2 Months

**FIGURE 3:**
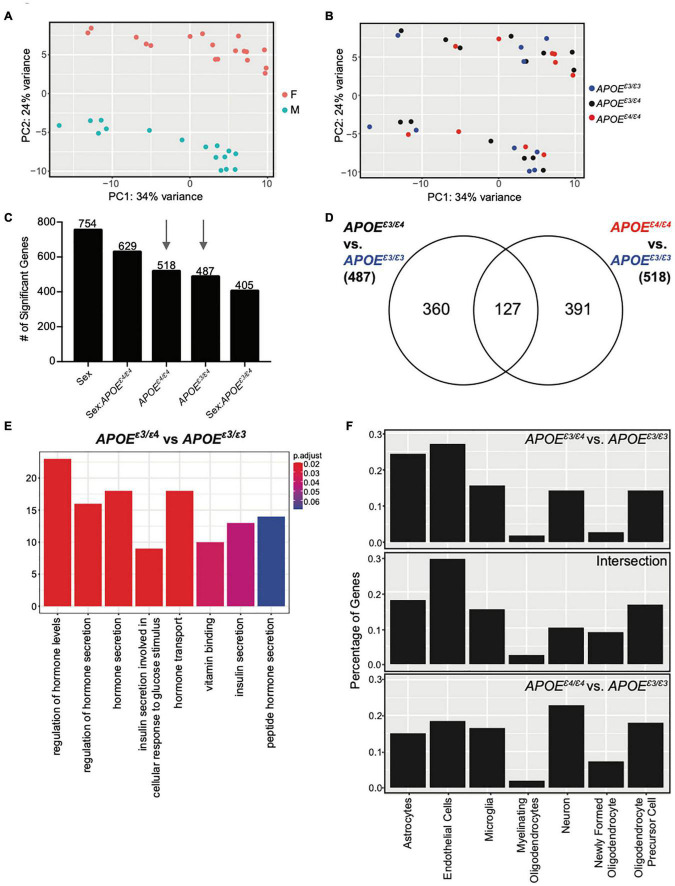
Cortical transcriptome analysis revealed unique functional enrichment for the *APOE^ε3/ε4^* genotype at 2 months. **(A)** PCA showed distinct clusters of male and female samples. **(B)** PCA showed samples do not cluster by *APOE* genotype at two mos. **(C)** Number of significant genes for each linear model predictor term (*p* < 0.05). **(D)** Number of significant genes unique to 2-month *APOE^ε4/ε4^* and *APOE^ε4/ε4^* when compared to *APOE^ε3/ε3^*, as well as the number of significant genes intersecting both lists. **(E)** Functional enrichment of the 360 significant genes (*p* < 0.10) unique to *APOE^ε3/ε4^* compared to *APOE^ε3/ε3^*. **(F)** Percentage of genes expressed by cell type according to the BrainRNAseq dataset.

We hypothesized that compared to *APOE^ε4/ε4^*, the *APOE^ε3/ε4^* genotype may differentially modulate cerebral processes. To assess this, RNA-seq was performed on cortical tissue collected from litter-matched male and female mice at 2 mos. Principal component analysis (PCA) showed clear separation between the sexes. There was no separation based on *APOE* genotype (Figures 3A,B) which agreed with previous studies (Zhao et al., 2020). Using *APOE^ε3/ε3^* as the ‘control’ genotype, linear modeling identified genes that varied as a function of sex, *APOE* genotype, and the interaction between sex and *APOE* genotype (Figure 3C, see the section “Materials and Methods”). Only 127 genes were in common between the 487 *APOE^ε3/ε4^* and the 518 *APOE^ε4/ε4^* genes (Figure 3D). GO terms enriched for the 360 unique *APOE^ε3/ε4^* genes included ‘regulation of hormone levels,’ ‘regulation of hormone secretion,’ and ‘insulin secretion’ (Figure 3E and Supplementary Figure 7). Hormone signaling, particularly in the glucose/insulin pathway, is disrupted in *APOE^ε4^* individuals and can increase the severity of dementia (Peila et al., 2002; Irie et al., 2008). No GO terms were enriched for either the 127 intersection genes or the 391 genes unique to *APOE^ε4/ε4^*, suggesting multiple unrelated processes may be more subtly affected. Interestingly, *APOE^ε4/ε4^* unique genes included angiogenesis-related *Acvrl1*, *Ang*, *Eng*, *Vegfc*, and cell adhesion-related *Fermt3*, *Itga2b*, *Itga5*. *Ang* can function by endothelial nuclear translocation to stimulate endothelial angiogenesis, while *Vegfc* is involved in vascular permeability (Moroianu and Riordan, 1994; Gao and Xu, 2008; Tammela et al., 2008; Miyake et al., 2015; Nagai and Minami, 2015; Apte et al., 2019). Both *Itga2b* and *Itga5* are receptors for fibrinogen and fibronectin, involved in the blood clotting cascade, while *Fermt3* is involved in leukocyte adhesion to endothelial cells (Varner et al., 1995; Oksala et al., 2015; Botero et al., 2020). RNA *in situ* hybridization (Allen Brain Atlas) and single cell sequencing of vascular-related cells confirm both *Acvrl1* (activin A receptor like Type 1) and *Eng* (endoglin) are expressed by vascular cells, including endothelial cells (Supplementary Figures 5, 6; Lein et al., 2007; Vanlandewijck et al., 2018).

Cell type analysis revealed that while the *APOE^ε3/ε4^* unique genes are expressed in multiple cell types, the majority of these genes are expressed by endothelial cells and astrocytes. In contrast, the majority of *APOE^ε4/ε4^* unique genes are expressed by neurons (Figure 3F). This further supports subtle but unique effects of *APOE* genotype to the cerebral cortex at a young age that may predispose for AD and dementia later in life.

### Sex and Physical Activity, but Not Apolipoprotein E Genotype Influence Peripheral Cholesterol Composition at 4 Months

**FIGURE 4:**
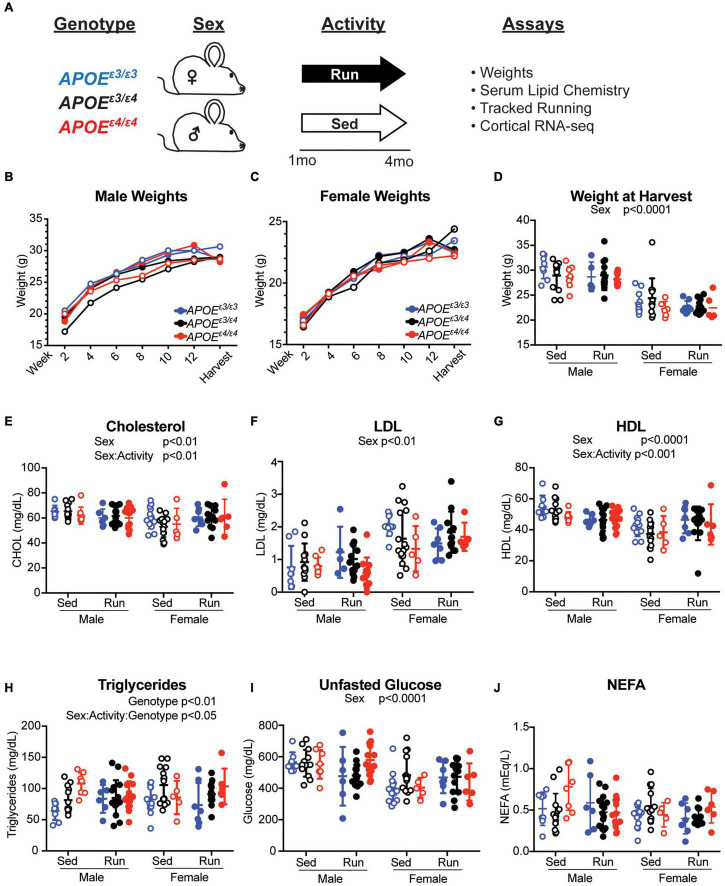
*APOE* genotype did not affect weight or cholesterol composition at 4 months. **(A)** Schematic of experiment and timeline. **(B,C)** Weights of male **(B)** and female **(C)** mice over the course of the running experiment. **(D)** Weight at harvest of both male and female mice across *APOE* genotypes showed a significant effect of sex. **(E)** Plasma total cholesterol concentrations revealed a main effect of sex and an interactive effect between sex and activity. **(F)** LDL concentration revealed a significant effect of sex. **(G)** HDL concentration revealed a significant effect of sex and interactive effect between sex and activity. **(H)** Triglyceride concentrations revealed a significant effect of *APOE* genotype and an interactive effect between sex, activity and *APOE* genotype. **(I)** Unfasted glucose concentrations revealed an effect of sex. **(J)** Non-esterified fatty acid (NEFA) concentrations revealed no significant effects. Significant ANOVA results are stated under the title of the graph, significant Tukey *post hoc* results can be found in Supplementary Table 2.

Physical activity, especially running, has been widely considered a prevention, reducing risk for age-dependent cognitive decline and dementia. However, exercise may not prevent dementia in all individuals. We hypothesized that this may be due in part to genetics. To begin to test this we determined whether the effects of exercise are influenced by *APOE* genotype in young mice. Litter-matched male and female *APOE^ε3/ε3^*, *APOE^ε3/ε4^* and *APOE^ε4/ε4^* mice were given access to a voluntary running wheel for 12 weeks from 1 mo to 4 mos (Figure 4A, see the section “Materials and Methods”). There was expected variation between mice for amount of time spent running and there were no differences in average rotations or running speed between *APOE* genotypes for both sexes, indicating activity may be compared across genotypes as it is not a confound (Supplementary Figures 8A–F). As has been shown before, female mice tended to run farther than male mice (Foley et al., 2019). As expected, there was a significant difference in weight between sexes at 4 mos, but there were no differences between the *APOE* genotypes or running and sedentary groups for either sex (Figures 4B–D).

Apolipoprotein E genotype did not significantly influence total cholesterol, LDL or HDL which agrees with previous findings in other *APOE* mouse models (Knouff et al., 1999; Mann et al., 2004). There was also no effect of *APOE* genotype on unfasted glucose, or NEFA concentrations in the plasma (Figures 4E–G,I,J). However, there was an effect of sex for total cholesterol, LDL, HDL and unfasted glucose levels, with males having higher total cholesterol, HDL, and unfasted glucose and lower LDL than females (Figures 4E–G,I). There was also an interaction between sex and activity for total cholesterol and HDL, suggesting the effect of activity on plasma cholesterol may be sex-specific at a young age (Figures 4E,G and Supplementary Table 2). There was a significant sex-activity-genotype interaction on triglyceride concentration (Figure 4H). Across total cholesterol and HDL concentration, there was an interesting sex-activity interaction, with activity appearing to have differential effects per sex. In total cholesterol and HDL there was an overall decrease in lipid concentration with running in males, however in females, there was an overall increase in these lipids. These results suggest that activity may have different effects on circulating lipid concentrations due to sex-based differences. Together, the effects of exercise on cholesterol composition were subtle and not influenced by *APOE* genotype, whereas triglyceride concentrations were influenced by sex, activity, and *APOE* genotype.

### Unique *APOE^ε3/ε4^* Genes Enrich for Extracellular Matrix (ECM)- and Coagulation-Related Terms

**FIGURE 5:**
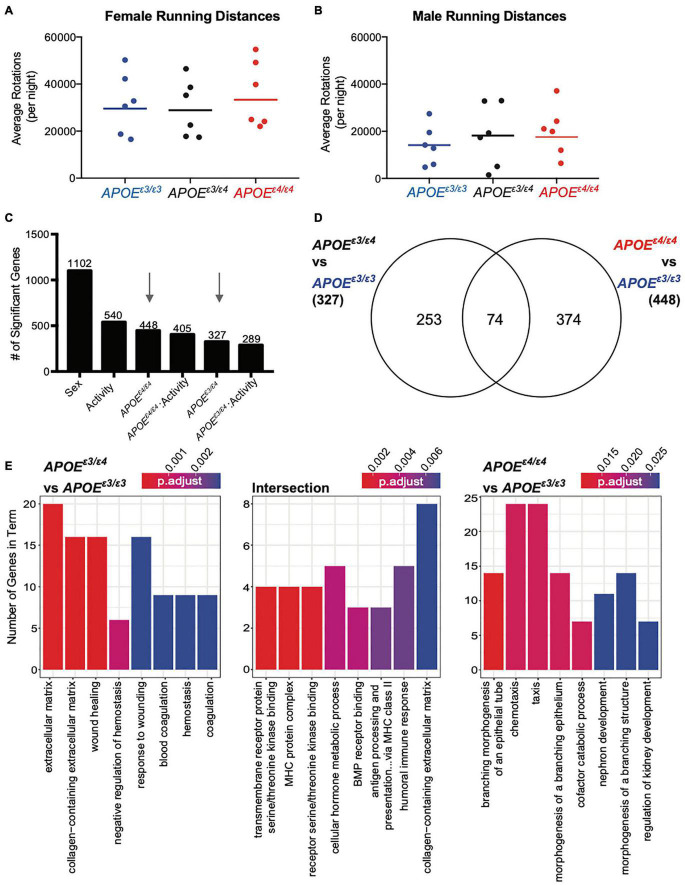
Significant genes unique for *APOE^ε3/ε4^* genotype showed functional enrichment for ‘extracellular matrix’ and ‘response to wounding’ at 4 months. **(A,B)** Average number of rotations per night during the dark cycle for female **(A)** and males **(B)** across all *APOE* genotypes for the six mice per group that were selected for RNA-seq. Mean is representative of all mice, including those that were not sequenced (see also Supplementary Figure 8). **(C)** Number of significant genes per each linear model predictor term. Red arrows highlight *APOE^ε4/ε4^* and *APOE^ε3/ε4^* terms used in panel **(D)**. **(D)** Number of significant genes unique to *APOE^ε3/ε4^* when compared to *APOE^ε3/ε3^*, and *APOE^ε4/ε4^* compared to *APOE^ε3/ε3^*, as well as the number of significant genes intersecting both groups. **(E)** Functional enrichment of the 253 genes unique to *APOE^ε3/ε4^* compared to *APOE^ε3/ε3^* (left), 374 genes unique to *APOE^ε4/ε4^* compared to *APOE^ε3/ε3^* (right) and 74 intersection genes between the two groups (middle) (*p* < 0.05).

To determine whether the effects of *APOE^ε4^* on the brain are modified by running in an *APOE* genotype-specific manner, cortical transcriptional profiling and linear modeling were performed (see the section “Materials and Methods”). Genes significantly associated with sex, *APOE^ε3/ε4^*, *APOE^ε4/ε4^*, activity and the interactions were identified (Figures 5A–C and Supplementary Figures 8C,D; Foley et al., 2019). The largest number of genes were significant for sex which enriched for ‘response to interferon beta,’ ‘response to virus,’ and ‘innate immune response,’ including known AD and dementia risk genes *Clu*, and *Plgc2*, suggesting a difference in immune function between the sexes (Supplementary Figure 10). There was no enrichment for the 540 genes significant for activity, suggesting multiple processes affected. Of the 540 genes, *Cx3cr1* and Csf1r were significant and have been previously implicated in maintaining microglia homeostasis, suggesting microglia response may be affected by activity across *APOE* genotypes (Keren-Shaul et al., 2017).

Of the 327 genes significant for *APOE^ε3/ε4^* and the 448 genes significant for *APOE^ε4/ε4^* only 74 intersected (22% from *APOE^ε3/ε4^* genes, 17% from *APOE^ε4/ε4^* genes) (Figure 5D) which was similar to our findings at 2 mos. Of the 253 genes unique for *APOE^ε3/ε4^* 63 genes were downregulated (negative β value) and 190 were upregulated (β value). The 253 *APOE^ε3/ε4^* unique genes enriched for GO terms such as ‘extracellular matrix,’ ‘collagen-containing extracellular matrix,’ and ‘wound healing’ (Figure 5E and Supplementary Figure 12A). The 374 *APOE^ε4/ε4^* unique genes enriched for ‘branching morphogenesis of an epithelial tube,’ ‘chemeotaxis’ and ‘taxis,’ including genes such as *Gli3*, *Cx3cr1*, and *Ccr1* (Figure 5C and Supplementary Figure 11A). Of the 374 genes there were 183 downregulated and 191 genes upregulated, indicated by a negative and a positive β-value, respectively. The 74 intersecting genes enriched for ‘MHC protein complex’ and ‘BMP receptor binding’ including genes associated with these GO terms included *Bmp5*, *Bmp6*, and *Spp1* (Supplementary Figure 11B). BMP (bone morphogenetic protein) signaling has been previously implicated in vascular development and more importantly, abnormal BMP protein expression can lead to vascular dysfunction, suggesting another pathway by which the *APOE^ε4^* allele influences AD and dementia pathologies (Garcia de Vinuesa et al., 2016). Of these 74 intersecting genes, 19 genes were downregulated and 55 genes upregulated, although the degree of regulation (β value) was different.

While many cell types can contribute to the extracellular matrix (ECM)- and coagulation-related pathways, endothelial cells contributed to 33% of the unique genes that were in the *APOE^ε3/ε4^* dataset (Supplementary Figure 12B). The cell types enriched for intersection genes included both oligodendrocyte precursor cells, as well as microglia (Supplementary Figure 12B). No specific cell types appeared to be responsible for *APOE^ε4/ε4^* unique genes. While there is no single cell type contributing to the *APOE^ε4/ε4^*-dependent transcriptional differences, endothelial cells may be critical drivers of transcriptional differences in *APOE^ε3/ε4^* mice (Supplementary Figure 12B). Interestingly, our data for *APOE^ε4/ε4^*-specific changes only minimally overlapped with previous studies (Supplementary Figure 13), differences likely due to dissimilarities in study design and environment (Zhao et al., 2020). The *APOE^ε3/ε4^* genotype was not studied previously (Zhao et al., 2020).

In examining genes unique to *APOE^ε3/ε4^* genotype and expressed by endothelial cells we observed several annexin genes. Both *Anxa1* and *Anxa2* contributed to the enrichment terms ‘extracellular matrix,’ ‘response to wound healing,’ and ‘coagulation.’ *Anxa2* has direct effects on the plasminogen/fibrin clotting response (Supplementary Figure 12C; Ling et al., 2004). While we expected a linear increase in gene expression with increasing *APOE^ε4^* gene dose, for endothelial-expressed *Anxa1*, *Anxa2*, and *Anxa3*, there was a significant increase in the *APOE^ε3/ε4^* genotype when compared to *APOE^ε3/ε3^*, but not a significant increase in the *APOE^ε4/ε4^* genotype when compared to *APOE^ε3/ε3^*. This pattern suggests that expression of some genes was differentially affected by the combination of the *APOE^ε3^* and *APOE^ε4^* allele.

To determine whether exercise effects the cortex in an *APOE* genotype-specific manner, we compared the genes for activity-*APOE^ε3/ε4^* with activity-*APOE^ε4/ε4^*. Only 17% of activity-*APOE^ε3/ε4^*, and 12% of activity-*APOE^ε4/ε4^* genes overlapped (Supplementary Figure 14A). Three intersection genes enriched for ‘anoikis’ (consisting of *Src*, *Tfdp1* and *Bmf*), suggesting a common cell death response between genotypes in response to activity (Supplementary Figures 14B,C). Together, these data show that exercise and *APOE* genotype do not appear to overtly interact on a transcriptional level in 4 mo mice.

### Weighted Gene Co-expression Network Analysis Confirms Apolipoprotein E Genotype-Specific Effects but Shows No Genotype by Activity-Specific Effects

**FIGURE 6:**
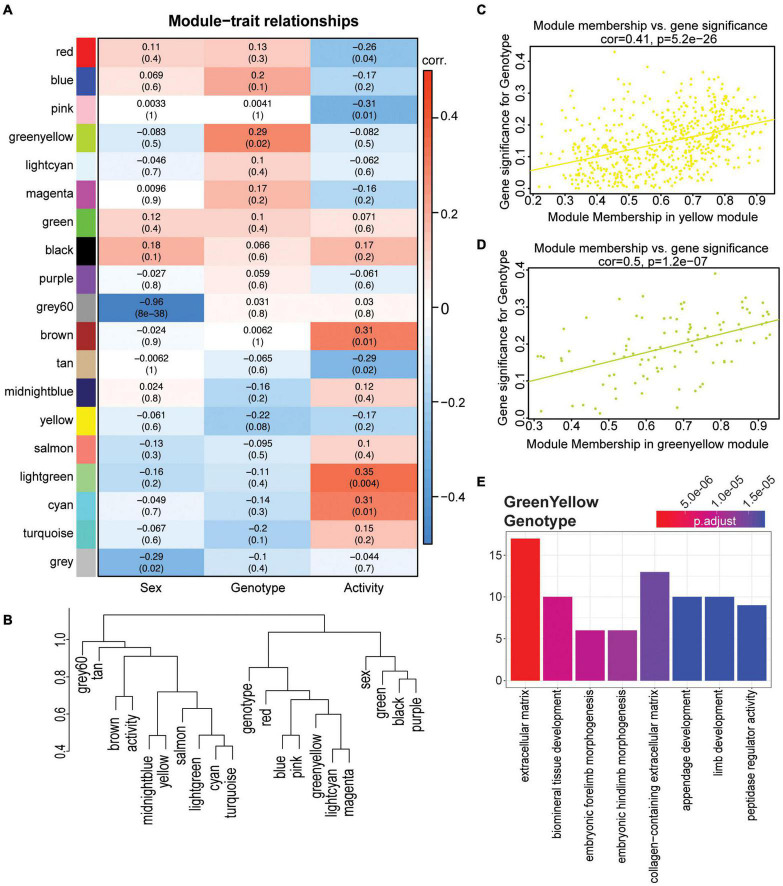
WGCNA confirmed functional enrichment of GO terms relating to extracellular matrix and morphogenesis is affected by *APOE* genotype. **(A)** Heatmap of module correlation and FDR values for sex, *APOE* genotype, and activity (*p* < 0.10). **(B)** Eigengene dendrogram of each module and sex, *APOE* genotype and activity. *Y* axis is a measure of dissimilarity. **(C,D)** Gene significance regressed against module membership to show correlation and significance for the yellow **(C)** and greenyellow **(D)** modules for *APOE* genotype. **(E)** Functional enrichment of the genes in the greenyellow module which is significant for *APOE* genotype (*p* < 0.05).

While linear modeling indicated subtle changes at the gene level, to ensure these changes were robust we employed a second approach, weighted gene co-expression network analysis (WGCNA) on the same 4 mo dataset. We used WGCNA to create modules of groups of genes across the entire dataset, thus reducing complexity of the large number of input genes to produce biologically relevant modules. This method differs from linear modeling by grouping genes into modules, including genes that may not be significant on the gene level, but still contribute to the coordination of a significant biological process. Nineteen modules were identified, and two modules were significantly correlated with sex (grey60, gray), two modules significantly correlated for genotype (yellow and greenyellow), and six modules significantly correlated for activity (cyan, lightgreen, tan, brown, pink, and red) (Figure 6A, see the section “Materials and Methods”). No modules were significant across multiple terms. Module eigengenes were calculated and dissimilarity plotted to visualize the relationship between modules and terms (Figure 6B). Both the yellow and greenyellow modules were significantly correlated to *APOE* genotype (Figures 6C,D). The yellow module showed no enrichment, however the genes in the greenyellow module enriched for ‘extracellular matrix,’ ‘embryonic morphogenesis,’ and ‘collagen-containing extracellular matrix’ and showed distinct differences between *APOE^ε3/ε3^* and both *APOE^ε3/ε4^* and *APOE^ε4/ε4^* (Figure 6C and Supplementary Figure 14D). Confirming our previous findings, these data suggest that *APOE* genotype affects the extracellular matrix, collagen, and morphogenesis (Figure 4).

The cyan and tan modules were significantly correlated for activity, and cyan enriched for ‘ensheathment of neurons,’ ‘myelination,’ and ‘gliogenesis,’ while the tan module enriched for ‘skeletal muscle cell differentiation,’ and ‘skeletal muscle organ development’ (Supplementary Figures 15A–D). However, no modules were significant for both *APOE* genotype and activity. These data support our previous findings by linear modeling that suggest the effects of exercise on cortical gene expression are not significantly impacted by *APOE* genotype, at least at this young age.

## Discussion

In this study, we demonstrated the importance of including the *APOE^ε3/ε4^* genotype to study the biology of APOE. *APOE^ε3/ε4^* is more common than *APOE^ε4/ε4^* across Caucasian, African America, Hispanic, and Japanese populations, which means there is a stark disconnect between genotypes evaluated in current mouse models to those present in human studies (Farrer et al., 1997). In human studies, the rarity of *APOE^ε4/ε4^* individuals results in a clustering of both *APOE^ε3/ε4^* and *APOE^ε4/ε4^* (often referred to as ‘*APOE^ε4^*+’ or ‘*APOE^ε4^* carriers’). These studies have been instrumental in understanding the effect of *APOE^ε4^* on cognitive health trajectories in humans, finding key biomarkers (e.g., soluble PDGFRβ) that are *APOE^ε4^*+ carrier specific, however the next step requires determining whether this is relevant for *APOE^ε3/ε4^* and *APOE^ε4/ε4^* similarly (Montagne et al., 2020). While informational, these strategies obfuscate differences between the two risk genotypes with the *APOE^ε3/ε4^* effect likely dominating the findings. In contrast, the vast majority of mouse studies performed to date primarily contrast the *APOE^ε4/ε4^* genotype with the *APOE^ε3/ε3^* genotype. Our transcriptome profiling approach predicts the *APOE^ε3/ε4^* genotype exerts unique effects on the cortex, therefore supporting more studies in mouse models to understand mechanisms by which the *APOE^ε3/ε4^* genotype increases risk for ADRDs. Our new humanized *APOE* mouse models and data help to facilitate these mechanistic studies.

Our results indicate that *APOE* genotype did not affect total cholesterol, HDL, or LDL levels in the blood at 2 and 4 mos. These data confirm reports using other humanized *APOE* models, where no *APOE* genotype differences were observed in plasma total cholesterol, HDL, or triglyceride in female mice between 2 and 5 mos (Knouff et al., 1999; Mann et al., 2004). It is of note that there are well-established differences in cholesterol composition between humans and mice due, at least in part, to a lack of cholesterol ester transfer protein (CETP) in mice which aids in the transfer of cholesterol esters depending on triglyceride concentration status (Davidson, 2010). This inability to properly transfer cholesteryl esters and triglycerides results in higher HDL and lower LDL levels in mice (Mabuchi et al., 2014). Cholesterol composition in heterozygous *APOE* genotypes throughout aging are still necessary, as this is when *APOE* genotype differences are reported to become more prominent.

Human studies have reported differences in triglycerides due to *APOE* genotype, with an increase in circulating triglyceride levels for each copy of *APOE^ε4^*. In our study, there was no *APOE* genotype effect on triglyceride levels at 2 mos, but differences were observed at 4 mos (Huang, 2010; Belloy et al., 2019). Data from other humanized *APOE* mice agreed with our 2 mo findings showing no significant difference in triglyceride concentrations between *APOE^ε3/ε3^* and *APOE^ε4/ε4^* mice (Knouff et al., 1999; Mann et al., 2004). While these findings indicate that *APOE* genotype does not affect lipid homeostasis at an early age, genotype-specific differences may come apparent at older ages – studies that are ongoing.

The *APOE^ε3/ε4^* genotype uniquely affected the cortical transcriptome at 2 and 4 mos (using *APOE^ε3/ε3^* as reference in our linear model). At 2 mos, genes unique for *APOE^ε3/ε4^* enriched for ‘regulation of hormone levels’ and ‘insulin secretion.’ At 4 mos, genes unique for *APOE^ε3/ε4^* enriched for ‘extracellular matrix,’ ‘collagen-containing extracellular matrix,’ and ‘blood coagulation,’ suggesting that the cerebrovasculature may be compromised. Our cell type analysis showed a greater contribution by endothelial cells in *APOE^ε3/ε4^* for these cerebrovascular terms, however this data is not inclusive of all cells in the cerebrovasculature, such as pericytes which have been shown to be impacted by *APOE* genotype (Montagne et al., 2020). Overall, these data supported our hypothesis that the *APOE^ε3/ε4^* risk genotype confers unique effects compared to *APOE^ε4/ε4^* and signify the need to better understand the effect of heterozygous *APOE* genotypes and how these differences may play a role in risk for dementia. Linear modeling is commonly used to identify significant changes to biological systems, and this is the case for the genotype differences identified in this study.

A previous study using other humanized *APOE* mice sought to examine early transcriptome and proteome changes in the *APOE ^ε4/ε4^*, *APOE ^ε3/ε3^* and *APOE ^ε2/ε2^* mouse cortex (Zhao et al., 2020). Analysis of their data with our linear modeling approach identified differences in the number of significant genes found between the two studies. We predict these differences are more likely due to study design, mouse cohort generation, mouse husbandry, environment, tissue harvesting, and sequencing platform rather than inherent differences between the humanized *APOE* models. However, future studies are necessary to compare the available humanized *APOE* models under the same conditions.

*APOE^ε4^* carrier females are at a greater risk dementia than their male counterparts. Postmenopausal females account for over 60% of AD cases (Riedel et al., 2016; Mosconi et al., 2018). In women, one *APOE^ε4^* allele causes an increase in dementia risk equivalent to two *APOE^ε4/ε4^* alleles in men (Riedel et al., 2016). Our study supports that sex-specific *APOE^ε4^* effects begin early in adolescence/adulthood and would be predicted to exacerbate dementia pathophysiology later in life. When examining the effect of sex by genotype interactions for *APOE^ε4^* status at 2 mos and then 4 mos, there were more genes significant for sex-*APOE^ε4/ε4^*, than sex-*APOE^ε3/ε4^*. These data indicate that *APOE* genotype interacts with sex-specific characteristics even at younger ages (as early as adolescence) to differentially predispose females and males to dementia later in life.

The molecular effects of exercise in the context *APOE* genotype are not known. With previous studies suggesting that up to a third of LOAD cases may be prevented by physical activity, it is of the utmost importance to understand the interactions between genetic risk and exercise (Norton et al., 2014). To elucidate the effects of exercise on dementia related pathology, most exercise-based murine studies utilized amyloidogenic models relevant to AD or middle cerebral artery occlusion models of VaD (Yuede et al., 2009; Otsuka et al., 2016; Choi et al., 2018; Khodadadi et al., 2018; Rezaei et al., 2018; Zhang et al., 2018). The few studies that have determined whether exercise is beneficial in the context of *APOE* utilized mostly *APOE* knockout mice (Nichol et al., 2009; Soto et al., 2015; Di Cataldo et al., 2016; Jakic et al., 2019; Zheng and Cai, 2019; Chaudhari et al., 2020). Our experimental design suggests exercise affects young mice in subtle ways that are not greatly impacted by *APOE* genotype, however aging studies may reveal greater *APOE* and exercise interactions. Linear modeling identified genes relevant to ECM health, the blood clotting cascade, development, and microglia homeostasis, all processes that play a role in modulating risk for ADRDs. Separately, WGCNA identified a module that correlated with activity which contained genes that enriched for myelination and gliogenesis (Supplementary Figure 15C). Previous studies have shown that voluntary exercise impacts myelination by increasing the proliferation oligodendrocyte precursor cells and mature oligodendrocytes in the motor cortex (Zheng et al., 2019).

Surprisingly, in our study, there were no significant functional enrichment terms for the genes unique to activity-*APOE^ε3/ε4^* and activity-*APOE^ε4/ε4^*. It will be important to understand whether longer term exercise paradigms are impacted by *APOE* genotype. *APOE^ε4^* exhibits harmful effects on the brain, predisposing for AD and cognitive decline. In general, few studies have determined whether long-term exercise is beneficial in the context of *APOE* risk genotypes. Exercise may mitigate or exacerbate *APOE^ε4^*-dependent risk depending on the specific biological process considered.

## Conclusion

These novel *APOE* models increase the ability to elucidate the mechanisms by which heterozygous *APOE* genotypes increase risk for AD and dementia. Our study predicts important unique effects of the *APOE^ε3/ε4^* genotype on AD-relevant phenotypes including biometrics and cortical gene expression. These differences need to be better understood to properly determine whether the mechanisms increasing risk for diseases such as AD and related dementias in those carrying one *APOE ^ε4^* allele are different from those carrying two, particularly as differential *APOE* genotype effects may be exacerbated at older ages. This work supports that research and therapies need to account for the impact of *APOE ^ε4^* allele dosage on ADRD risk and pathology.

## Data Availability Statement

The data presented in the study are publicly available via www.synapse.org, Synapse ID:syn26561824.

## Ethics Statement

The animal study was reviewed and approved by Animal Care and Use Committee at The Jackson Laboratory.

## Author Contributions

MS, GC, and GH designed *APOE* mouse models. KF and GH conceived and designed this project and wrote and prepared this manuscript. AH, DG, KK, and KF validated the *APOE* mouse models. KF performed mouse experiments, data collection, and bioinformatic analysis. KF, GC, and GH consulted for statistical approach and analysis. All authors read and approved the final manuscript.

## Conflict of Interest

The authors declare that the research was conducted in the absence of any commercial or financial relationships that could be construed as a potential conflict of interest.

## Publisher’s Note

All claims expressed in this article are solely those of the authors and do not necessarily represent those of their affiliated organizations, or those of the publisher, the editors and the reviewers. Any product that may be evaluated in this article, or claim that may be made by its manufacturer, is not guaranteed or endorsed by the publisher.

## Abbreviations

LOAD: late onset Alzheimer’s disease
VaD: vascular dementia
HDR: homology directed recombination
VLDL: very low density lipoprotein
LDL: low density lipoprotein
HDL: high density lipoprotein
NEFA: non-esterified fatty acids
WGCNA: weighted gene co-expression network analysis
GO: gene ontology.
