# Supplementary material for: The APOEε3/ε4 Genotype Drives Distinct Gene Signatures in the Cortex of Young Mice
Source: Front Aging Neurosci. 2022 Mar 16;14:838436. doi: 10.3389/fnagi.2022.838436 (PMC8967347; doi:10.3389/fnagi.2022.838436)
Supplement: Supplementary file 1 [file Data_Sheet_1.docx]

**Supplemental Figure 1.**

**A.** Schematic of previously established APOE mouse models. **B.** Mouse *Apoe* region across controls and humanized *APOE^ε4^* mice show removal of mouse Apoe sequence on mouse chromosome 7. **C.** Humanized *APOE^ε4^* mice have the human sequence that aligns with human *APOE* on human chromosome 19.

**Supplemental Figure 2.**

Full western blots of Mouse Apoe (A), pan Human APOE (B), Human APOE^ε4^ (C), and Actin (D). Specific bands labeled in E.

**Supplemental Figure 3.**

Full western blots of quantified APOE^ε4^ expression in *APOE^ε3/ε3^*, *APOE^ε3/ε4^* and *APOE^ε4/ε4^* male and female mice from Fig 1D. M = Male, F = Female. *APOE^ε4^* molecular weight is 34kDa, Actin molecular weight is 42kDa.

**Supplemental Figure 4.**

Cholesterol levels in brain tissue of male and female *APOE^ε3/ε3^*, *APOE^ε3/ε4^* and *APOE^ε4/ε4^* mice at 2 mo.

**Supplemental Figure 5.**

Cortical gene expression for key genes in *APOE^ε3/ε3^*, *APOE^ε3/ε4^* and *APOE^ε4/ε4^* mice.

**Supplemental Figure 6.**

Gene expression (A,D), Allan Brain Atlas *in situ* cortical expression (B,E), and cell specific brain expression (C,F) adapted from Vanlandewijck et al. 2018, for key genes *Acvrl1* and *Eng*.

**
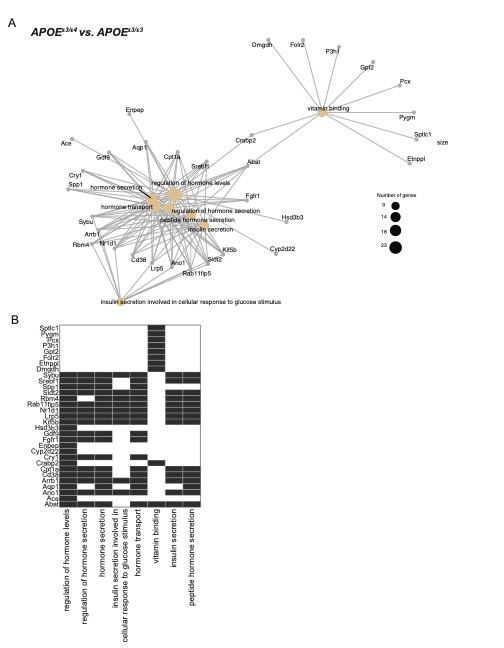
**

**Supplemental Figure 7.**

**A.** Significant genes contributing to unique 2 month *APOE*^ε3/ε4^ functional enrichment terms. **B.** Genes present in *APOE*^ε3/ε4^ functional enrichment terms.

**Supplemental Figure 8.**

**A and B.** Percentage of time spent running for female (A) and males (B) for each *APOE* genotype. **C and D.** Average number of rotations per night during the dark cycle for all mice tracked for female (C) and males (D) across all *APOE* genotypes. Black outlined dots represent mice selected for transcriptional profiling. **E and F.** Percentage of time spent running at different speeds (measured at rotations per minute) for females (E) and males (F) across all *APOE* genotypes.

**Supplemental Figure 9.**

**A** Representative image of NeuN and DAPI staining in a 4 month female sedentary cortex. **B.** IMARIS colocalization rendering of NeuN and DAPI. **C.** IMARIS colocalization used for spot detection and quantification of NeuN+DAPI+ cells analysis. **D.** Quantification of NeuN+DAPI+ cell spots show no significant difference by One-way ANOVA across *APOE* genotypes.

**
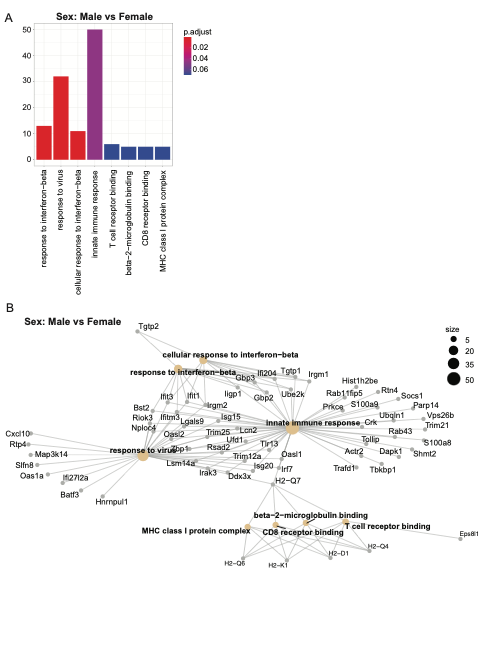
**

**Supplemental Figure 10.**

**A.** Functional enrichment of the genes significant for sex in our linear model. **B.** Significant genes for 4 month sex enrichment terms.

**
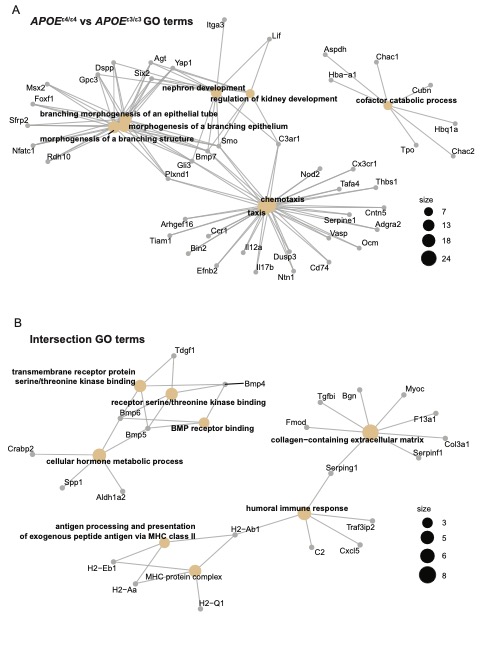
**

**Supplemental Figure 11.**

**A.** Significant genes contributing to the functional enrichment terms *APOE^ε4/ε4^* compared to *APOE^ε3/ε3^*. **B.** Significant genes contributing to the functional enrichment terms in the interaction between *APOE^ε3/ε4^* compared to *APOE^ε3/ε3^* and *APOE^ε4/ε4^* compared to *APOE^ε3/ε3^*.

**
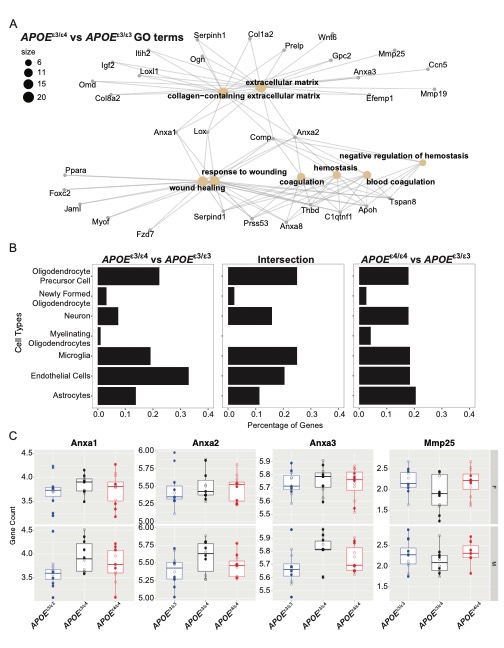
**

**Supplemental Figure 12.**

**A.** Significant genes contributing to unique *APOE*^ε3/ε4^ functional enrichment terms. **B.** Cell specific expression (expressed as a percent of total genes queried) of 253 significant genes unique to *APOE*^ε3/ε4^ compared to *APOE*^ε3/ε3^ (left), 74 significant genes in the intersection between the two groups (middle), and 374 significant genes unique to *APOE*^ε4/ε4^ compared to *APOE*^ε3/ε3^ (right). **C.** Gene expression of significant genes for *APOE*^ε3/ε4^ compared to *APOE*^ε4/ε4^ expressed by endothelial cells (Anxa1 p=0.012477815; Anxa2 p= 0.013759677, Anxa3 p= 0.043613649, Mmp25 p= 0.009623381)

**Supplemental Figure 13.**

**A.** Linear modeling results on Zhao 3-month data, our 2-month-old data, and 4 month data. **B.** PCA of Zhao 3-month data for colored for sex and *APOE* genotype. **C.** PCA of 2 month samples colored for sex and *APOE* genotype. **D.** PCA of 4 month data for colored for sex and *APOE* genotype. **E.** Zhao 3 month enrichment for the significant genes for sex, *APOE*^ε4^, and sex-*APOE*^ε4^ interaction.

**
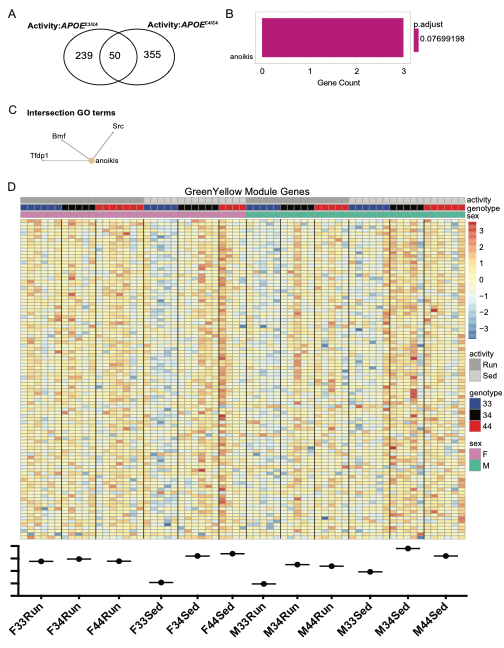
**

**Supplemental Figure 14.**

**A.** Number of significant genes for the interaction between activity and *APOE*^ε3/ε4^, the interaction between activity and *APOE*^ε4/ε4^, and the intersection. **B.** Functional enrichment of the genes significant for the intersection between activity and *APOE*^ε3/ε4^ and activity and *APOE*^ε4/ε4^. **C.** Significant genes for anoikis enrichment terms. **D.** Heatmap of gene expression for genes in the GreenYellow module and average expression per sex/genotype/activity group.

**Supplemental Figure 15.**

**A and B.** Gene significance regressed against module membership to show correlation and significance for the cyan (A) and tan (B) modules for activity. **C.** Functional enrichment of the genes in the cyan module which is significant for activity. **D.** Functional enrichment of the genes in the tan module which is significant for activity.

**Supplemental Table 1.**

2 mo Cholesterol Composition ANOVA Results.

**Supplemental Table 2.**

4 mo Cholesterol Composition ANOVA Results.
